# An alternate route to phosphorylating DegU of *Bacillus subtilis* using acetyl phosphate

**DOI:** 10.1186/s12866-015-0410-z

**Published:** 2015-03-31

**Authors:** Lynne S Cairns, Jessica E Martyn, Keith Bromley, Nicola R Stanley-Wall

**Affiliations:** Division of Molecular Microbiology, College of Life Sciences, University of Dundee, Dundee, DD1 5EH UK; Current address: Department of Molecular Biology and Microbiology, Tufts University School of Medicine, Boston, MA 02111 USA; Current address: Sir William Dunn School of Pathology, South Parks Road Oxford, Oxford University, Oxford, OX1 3RE UK; James Clerk Maxwell Building, School of Physics, University of Edinburgh, Edinburgh, EH9 3JZ UK

**Keywords:** *Bacillus subtilis*, DegU, Acetyl phosphate, Biofilm, Swarming

## Abstract

**Background:**

Two-component signal transduction pathways allow bacteria to sense and respond to the environment. Typically such pathways comprise a sensor histidine kinase and a response regulator. Phosphorylation of the response regulator commonly results in its activation, allowing the protein to bind to target promoter elements to regulate transcription. Several mechanisms are used to prevent inappropriate phosphorylation of the response regulator, thereby ensuring a specific response. In *Bacillus subtilis*, the DegS-DegU two-component system controls transcription of target genes in a manner dependent on the level of the phosphorylated response regulator, DegU. Previous work has tentatively indicated that DegU, and DegU H^12^L, a DegU variant which displays enhanced stability of the phosphoryl moiety, can be phosphorylated in the absence of the kinase, DegS*.*

**Results:**

The data presented here reveal that DegU H^12^L requires aspartic acid 56 (D^56^), the identified DegU phosphorylation site, for its activity. By indirectly measuring the level of DegU ~ P in the cell by assessment of several well recognised DegU regulated processes it was shown that DegU H^12^L retains its activity in the absence of DegS, and that mutation of D^56^ produced an inactive protein. Further experiments designed to raise the level of acetyl phosphate within the cell suggest that DegU can be phosphorylated by acetyl phosphate in the absence of *degS*. Additionally, the phenotypic and biochemical experiments presented indicate that DegU H^12^L can reliably mimic high levels of phosphorylated DegU.

**Conclusions:**

The ability of acetyl phosphate to modify DegU, and indeed DegU H^12^L, reveal an additional layer of regulation for DegU phosphorylation that will be relevant when the level of DegS is low or in the absence of *degS*. Given the number of processes that DegU can activate or inhibit, extensive regulation at a number of levels is required to ensure that the system is not inappropriately stimulated. DegS has both kinase and phosphatase activity and our findings demonstrate that the phosphatase activity of DegS is essential to control the level of DegU phosphate. Overall we contribute to our understanding of how the intricate signalling pathway DegS-DegU is regulated in *B. subtilis*.

## Background

Signal transduction pathways are used by bacteria to sense and respond to their local environment. Two-component signal transduction systems (TCS) are a prototypical signalling cascade that bacteria use to couple changes in the extracellular environment to physiological effects. The signals that activate TCS are highly varied and include cell shape [1], temperature [2], osmolarity [3], cellular redox state [4] and quorum signals [5]. The responses that are elicited are similarly diverse and encompass biofilm formation, motility, activation of secretion systems and secretion of virulence factors [6]. These responses all share the common theme of promoting survival of the individual cell, or indeed of the population.

TCS typically comprise a sensor histidine kinase (HK), which receives a signal and a cognate response regulator (RR), which elicits the effect. The HK consists of an input domain, which can detect a signal, and a kinase domain. Upon perception of a signal the HK autophosphorylates [7]. The phosphoryl group is transferred to a conserved aspartic acid residue on the N-terminal receiver domain of the RR, resulting in a conformational change in the output domain of the RR [8]. The RR typically elicits downstream effects by binding to DNA to alter gene expression [9], although some RRs have been shown to have intrinsic enzymatic activity or to impact physiological processes by their interaction with other proteins [6]. Bacteria use a number of mechanisms to ensure that distinct pathways are insulated from inappropriate crosstalk [7]. Such specificity in the response may be engineered by additional proteins or solely by the HK and RR themselves. For example, HKs can exhibit dual kinase and phosphatase activities and many HKs have higher affinities for their cognate RR than for any other RR in the cell [7].

The genome of the Gram-positive soil bacterium *Bacillus subtilis* encodes for 36 histidine kinases and 34 response regulators [10]. These signal transduction pathways are involved in regulating a number of diverse cellular processes that allow *B. subtilis* to efficiently adapt to its surroundings. One such TCS is DegS-DegU, where DegS is a cytoplasmic sensor histidine kinase and DegU is the cognate response regulator [11-13]. The accessory protein DegQ aids the transfer of the phosphoryl group to DegU by stabilising phosphorylated DegS [14]. Given that DegS is cytoplasmic it is likely that DegQ engineers additional specificity into the system to prevent DegS from phosphorylating non-cognate RRs. DegS also has intrinsic phosphatase activity [15]. The activity of the DegS-DegU TCS is subject to extensive regulation at both the transcriptional and post-translational levels (for a review see [16]). The signal that activates DegS-DegU has remained somewhat elusive, but recent work has identified the ability of DegS-DegU to sense rotation of the flagellum, where impedance of rotation results in increased levels of phosphorylated DegU (DegU ~ P) [17].

Together DegS and DegU regulate a myriad of cellular processes by impacting the transcription of target genes. This is a complex process as the genes regulated differ depending on the level of phosphorylated DegU (DegU ~ P) within the cell [18,19]. For example, unphosphorylated DegU is required for genetic competence [20], low levels of DegU ~ P are required for swarming motility [18,19], mid-levels promote biofilm formation [18,19] and high levels are needed for the production of exoproteases [15] and of the exopolymer γ-poly-D-L-glutamic acid (hereafter γ-PGA) [21]. Furthermore, high levels of DegU ~ P inhibit swimming and swarming motility, and biofilm formation [22,23]. The activity of DegU ~ P has been linked with the presence of a small protein called SwrA [24]. It is proposed that SwrA enhances the ability of DegU ~ P to bind to the *fla/che* promoter, which drives the transcription of the major flagellar gene operon [24]. This model suggests that DegU ~ P functions as a transcriptional activator in the presence of SwrA, but shows repressive effects in its absence.

The ability of DegU ~ P to modulate such a broad range of processes has been exploited to further understand the intricacies of the regulatory system. Aspartic acid phosphorylation is an extremely labile modification and as such it is difficult to detect *in vivo* or *ex vivo* [25]. Thus, the level of DegU ~ P is typically inferred by measurement of the impact on the transcription of target genes, or by assessing the up- or down-regulation of specific physiological processes [17,18]. Understanding of the mechanisms behind the regulation of DegU ~ P controlled processes has been aided by the use of several point mutations in the coding regions of both *degS* and *degU* that have been harnessed to test the effects of *(i)* inhibiting the phosphorylation of DegU or *(ii)* promoting phosphorylation of DegU. For example, the *degU (D56N)* (also known as *degU146)* allele encodes for the DegU D^56^N protein variant where the aspartic acid phosphorylation site is mutated to asparagine [11,26]. Additionally other studies use the *degU (H12L)* (also known as *degUhy32*) allele where histidine at position 12 is mutated to leucine (DegU H^12^L) [27]. This variant of DegU exhibits a dephosphorylation rate that is seven times slower than that of phosphorylated wild-type DegU [26] and has therefore been used to test the effects of increasing levels of DegU ~ P on multiple DegU controlled outputs [18,19,23]. However it is important to note that recent work has suggested that an inability of DegU H^12^L to interact with the SwrA protein may explain its inhibitory effect on flagellar motility, rather than this being the result of high levels of DegU ~ P *per se*. The authors suggest that motility is retained when the levels of wild-type DegU ~ P are elevated using variants of DegS that lack dephosphorylation activity. Therefore it was concluded that use of the DegU H^12^L variant may not directly mimic high levels of DegU ~ P in the cell [24].

Previous studies have suggested that DegS may not be essential for the phosphorylation of DegU *in vivo* [19]. Evidence for this was provided by experiments where strains lacking *degS* or carrying the *degU (H12L)* allele were examined. For instance, deletion of *degU* or mutation of the DegU phosphorylation site (D^56^N) does not permit swarming motility [18], suggesting that DegU ~ P is required for this multicellular behaviour. However, a strain that lacks *degS* is still able to swarm [18]. Therefore, while DegU ~ P is needed for swarming motility, the kinase DegS is not. Additional preliminary data indicate that when *degU (H12L)* (encoding DegU H^12^L) is expressed in the absence of DegS γ-PGA production is still observed [17]; this is a phenotype associated with high levels of DegU ~ P [21]. The aim of this work was to ascertain if DegU and DegU H^12^L could be phosphorylated by an alternative means in strains that lacked *degS* and, if so, how this occurred. Here a combination of bacterial genetics and phenotypic assays using the undomesticated *B. subtilis* NCIB3610 strain [28] reveal that DegU H^12^L requires phosphorylation for its activity. These data also suggest a role for the small molecule phospho-donor acetyl phosphate (AcP) in phosphorylating DegU and DegU H^12^L in the absence of *degS*. Experiments designed to test the effect of artificially raising the level of AcP through genetic manipulation revealed an increase in exoprotease production and an inhibition of motility. Overall this work highlights an alternative means by which the phosphorylation, and therefore activity, of DegU can be regulated and furthermore provides evidence to suggest that DegU H^12^L can accurately mimic high levels of DegU ~ P in the cell.

## Methods

### Growth conditions and strain construction

*Escherichia coli* and *Bacillus subtilis* strains were routinely grown in Lysogeny broth (LB) (10 g NaCl, 5 g yeast extract, 10 g tryptone per litre) or on LB plates supplemented with 1.5% select agar (Invitrogen) at 37°C unless otherwise stated. When appropriate, isopropyl β-D-1-thiogalactopyranoside (IPTG) was added at the indicated concentrations. As required *B. subtilis* strains were grown in MSgg medium (5 mM potassium phosphate and 100 mM MOPs at pH 7.0 supplemented with 2 mM MgCl_2_, 700 μM CaCl_2_, 50 μM MnCl_2_, 50 μM FeCl_3_, 1 μM ZnCl_2_, 2 μM thiamine, 0.5% glycerol, and 0.5% glutamate). *E. coli* strain MC1061 [*F*’*lacIQ lacZM15 Tn10* (*tet*)] was used for the routine construction and maintenance of plasmids. When required, antibiotics were used at the following concentrations: 100 μg ml^−1^ ampicillin, 100 μg ml^−1^ spectinomycin, 25 μg ml^−1^ kanamycin, 1 μg ml^−1^ erythromycin and 25 μg ml^−1^ lincomycin. Strains were constructed using standard protocols. Phage transductions were carried out as previously described (Verhamme *et al*., [18]). Strains, plasmids and primers used in this work are listed in Tables 1, 2 and 3, respectively.

**Table 1:** ***Bacillus subtilis*** **strains used in this study**

**Strain**	**Relevant genotype** ^**a**^	**Source** ^**b**^
**NCIB3610**	*Bacillus subtilis* Prototroph	B.G.S.C.
**168**	*Bacillus subtilis trpC2*	B.G.S.C.
**JH642**	*Bacillus subtilis trpC2 pheA1*	[50]
**TMP147**	168 *ackA::mls*	[48]
**DS1677**	3610 Δ*hag*	D. Kearns
**QB136**	*leuB8 trpC2 degU32-hy*	[30] B.G.S.C
**QB4144**	168 *degU146*	[12]
**NRS1136**	JH642 *degS::pDH64 (cml)*	[18]
**NRS1183**	JH642 Δ*degSU::spc*	[21]
**NRS1287**	JH642 *amyE::P*_*hyspank*_ *-degU-32-hy-lacI (spc)*	[18]
**NRS1314**	3610 *degU::pBL204 (cml)*	[18]
**NRS1325**	3610 *degU::pBL204 (cml) amyE::*P_*hy-spank*_ *-degU32-hy-lacI (spc)*	[18]
**NRS1326**	3610 *degU::pBL204 (cml) amyE::*P_*hy-spank*_ *-degU-lacI (spc)*	[18]
**NRS1327**	3610 *degU::pBL204 (cml) amyE::*P_*hy-spank*_ *-degU146-lacI (spc)*	[18]
**NRS1358**	3610 *degS::pDH64 (cml)*	[18]
**NRS1433**	JH642 *pgsB::pBL141 (spc)*	[21]
**NRS1499**	3610 Δ*degSU::spc*	SPP1 NRS1183 → NCIB3610
**NRS4419**	3610 Δ*degSU::spc amyE::*P_*hy-spank*_ *-degU32-hy-lacI (spc)*	SPP1 NRS1287 → NRS1499
**NRS4756**	168 *amyE::*P_*spac*_-*degU32hy-degU146-lacI (cml)*	pNW1057 → 168
**NRS4761**	3610 Δ*degSU::spc amyE::*P_*spac*_ *-degU32-hy-degU146-lacI (cml)*	SPP1 NRS4756 → NRS1499
**NRS4762**	3610 *degU::mls amyE::*P_*spac*_ *- degU32-hy-degU146-lacI (cml)*	SPP1 NRS4756 → DS1993
**NRS4770**	3610 *ackA::mls*	SPP1 TMP147 → NCIB3610
**NRS4771**	3610 *ackA::mls degS::pDH64 (cml)*	SPP1 TMP147 → NRS1358
**NRS4819**	3610 *degS::cml ackA::mls pgsB::spc*	SPP1 NRS1443 → NRS4771

^a^Drug resistance cassettes are indicated as follows: *mls*, lincomycin/erythromycin resistance; *cml*, chloramphenicol resistance and *spc*, spectinomycin resistance. All JH642 strains also carry *trpC2* and *pheA1* mutations. Note that the *degU32-hy* allele encodes DegU H^12^L and the *degU146* allele encodes DegU D^56^N.

^b^The direction of strain construction is indicated with DNA or phage (SPP1) (→) recipient strain.

B.S.G.C. is the *Bacillus* genetic stock centre.

**Table 2:** **Plasmids used in this study**

**Plasmid**	**Relevant genotype**	**Source**
**pPL82**	*amyE integration plasmid*	[29]
**pQE60**	*His6 protein purification plasmid*	Qiagen
**pNW5**	*degU (H* ^*12*^ *L)* in pPL82	This study
**pNW43**	*degU in* pQE60	[18]
**pNW52**	*degU (D* ^*56*^ *N) in* pQE60	This study
**pNW54**	*degU (H* ^*12*^ *L) in* pQE60	This study
**pNW1057**	*degU (H* ^*12*^ *L, D* ^*56*^ *N)* in pPL82	This study
**pNW1072**	*degU (H* ^*12*^ *L, D* ^*56*^ *N) in* pQE60	This study

**Table 3:** **Primers used in this study**

**Primer**	**Target**	**Sequence** ^**a**^
**NSW10**	*degU (H* ^*12*^ *L)*	GCTAGAGTATATA*AAGCTT*GAACAATAATACAAGGAG
**NSW11**	*degU (H* ^*12*^ *L)*	GCCTAAAAAAA*GCATGC*GACCTGCCTAGTAAAAGG
**NSW79**	*degU*	CGTGGC*CCATGG*CTAAAGTAAACATTG
**NSW80**	*degU*	ATA*AGATCT*CATTTCTACCCAGCC
**NSW1649**	*degU*	TGATGTTGTGATCATG***AAT***ATCAATATGCCAAACG
**NSW1650**	*degU*	CGTTTGGCATATTGAT***ATT***CATGATCACAACATCA

^a^engineered restriction sites are indicated by italics and nucleotides for mutagenesis are highlighted in italic bold text.

### Plasmid construction

#### Construction of pNW5

Plasmid pNW5, used as a template for the production of pNW1057, is a derivative of pPL82 [29]. Primers NSW10 and NSW11 were used to amplify *degU (H12L)* from the chromosome of strain QB136 [30]. The PCR product was digested with HindIII and SphI using restriction sites engineered into the primers (Table 3) and cloned into pPL82 cut the same.

#### Construction of pNW52 and pNW54

Plasmids pNW52 and pNW54, used to over-produce the DegU protein variants DegU D^56^N and DegU H^12^L, were constructed in a similar manner to pNW43 [18]. However the primers NSW79 and NSW80 were used to amplify the *degU* coding region from genomic DNA harvested from strains QB4414 [12] or QB136 [30], respectively. The PCR products were digested with NcoI and BglII using restriction sites engineered into the primers and cloned into pQE60 cut the same.

#### Construction of pNW1057

Plasmid pNW1057, used to introduce the *degU (H12L)-degU (D56N)* coding region under the control of the IPTG inducible promoter P_*spac*_ at the non-essential *amyE* locus, is a derivative of pNW5. The *degU (D56N)* mutation (resulting in the mutation of aspartic acid at position 56 to asparagine; D^56^N) was introduced to pNW5 by site-directed mutagenesis with primers NSW1649 and NSW1650 using KOD Hot Start DNA Polymerase (Novagen) followed by DpnI digestion. The resulting DNA was transformed into competent *E. coli* MC1061 cells and the presence of the mutation confirmed by DNA sequencing.

#### Construction of pNW1072

Plasmid pNW1072, used to over-produce the DegU H^12^L D^56^N protein variant, is a derivative of pNW54 and was constructed in a similar manner to pNW1057, using pNW54 as a template in the site-directed mutagenesis PCR reaction.

### Motility assays

Swimming and swarming analyses were performed as described before (Verhamme *et al*., [18]) using low-salt LB (5 g NaCl, 5 g yeast extract, 10 g tryptone per litre) supplemented with 0.4% or 0.7% Bacto agar (Invitrogen), respectively. Plates were incubated at 37°C and the extent of swimming or swarming noted at defined intervals.

### Protease plate assays

Analysis of protease production was carried out as previously described (Verhamme *et al*., [18]). Briefly, secreted protease production was analysed using LB agar plates supplemented with 1.5% (w/w) dried milk powder. *B. subtilis* cultures were grown to mid-late exponential phase in LB and 10 μl of culture spotted on to each plate (containing IPTG as required) and incubated at 37°C for 18 h prior to being photographed.

### Western blot

To extract proteins for Western blot analysis cells were grown as lawns overnight at 20°C on LB medium supplemented with 1.5% agar. Cells were diluted to an OD_600_ of 0.01 and inoculated into MSgg medium, containing IPTG as required, and grown at 37°C with aeration. When cultures reached the stationary phase of growth cells were collected by centrifugation at 4700 *x g*. Cell pellets were suspended in SDS loading dye, normalised to OD_600_, separated by SDS-PAGE and transferred onto PVDF membrane (Millipore) by electroblotting. Antibodies raised against DegU [18] and rabbit anti-sheep HRP conjugated secondary antibodies were used at a dilution of 1:5,000 (Pierce).

### Complex colony morphology assay

Complex colony morphology assays were performed as previously detailed [28,31]. Essentially 10 μl of liquid LB cultures grown to mid-exponential phase were spotted onto MSgg medium solidified with 1.5% agar and incubated at 30°C for 48 hours prior to imaging using a Leica MZ16 stereoscope.

### Purification of DegU protein variants

DegU-His_6_ protein variants were purified using a method based on that used by Verhamme *et al*. [18]. Plasmids pNW43, pNW52, pNW54 and pNW1072 were transformed into M15 pRep4 *E. coli* cells. Cells carrying each plasmid were grown in starter cultures of LB to mid-exponential phase and diluted back to an OD_600_ of 0.01 in 1 L of LB media supplemented with ampicillin and kanamycin and grown at 37°C with aeration to mid-exponential phase. Protein production was induced by the addition of 50 μM IPTG and cells were grown overnight at 26°C with aeration. Cells were harvested by centrifugation (4000 *x g* for 45 min) and washed twice in purification buffer (25 mM Tris–HCl pH 8, 250 mM NaCl, 10 mM imidazole) supplemented with complete EDTA-free protease inhibitor tablets (Roche). Cells were lysed using an Emulsiflex cell disruptor by applying 15,000 psi of pressure three times to each sample. Cell debris was removed by centrifugation (27,000 *× g*, 20 min) and the supernatant applied to pre-cleared Ni-NTA agarose beads (Qiagen) (1.5 ml of beads per 1 L of culture) and incubated at 4°C with gentle agitation for 2 h to allow the His tagged protein to bind to the beads. The lysate was loaded onto a 25 ml gravity flow column (Bio-Rad) and allowed to flow-through. The beads were washed twice with wash buffer (purification buffer supplemented with 30 mM imidazole) and eluted with increasing concentrations of imidazole, up to 250 mM. Protein purity was assessed by SDS-PAGE and proteins were concentrated using VivaSpin concentrators (Sartorius). Proteins were further purified by size-exclusion chromatography using a Superdex 75 10/300 GL column (GE Healthcare). Fractions containing pure DegU were combined and concentrated with VivaSpin concentrators and exchanged into 25 mM phosphate buffer for circular dichroism experiments.

### Circular dichroism analysis

Circular dichroism (CD) was performed using a Jasco J-810 spectropolarimeter. Samples were analysed at a concentration between 0.1 – 0.2 mg.mL^−1^ (4 – 8 μM) in a 0.1 cm quartz cuvette. Measurements were performed with a scan rate of 50 nm.sec^−1^, a data pitch of 0.1 nm and a digital integration time of 1 sec. Twenty accumulations were measured and averaged to produce the final curve.

## Results and discussion

### The sensor kinase DegS is dispensable for activation of the DegU protein variant, DegU H^12^L

Previous observations indicate that the sensor kinase DegS may be dispensable for the phosphorylation of DegU [18,19] and the DegU protein variant, DegU H^12^L (encoded by the *degU (H12L)* allele) [17]. To extend and confirm these findings we utilised strains where the *degU (H12L)* coding region was integrated at a non-essential location under the control of the IPTG inducible promoter P_*hy-spank*_ on the chromosome of strains that carried either an insertion in the native *degU* gene (resulting in NRS1325), or a deletion of both the *degS* and *degU* coding regions (resulting in NRS4419) (see Table 1). Three different biological assays were used to (indirectly) assess the activity of DegU H^12^L.

**Figure 1:**
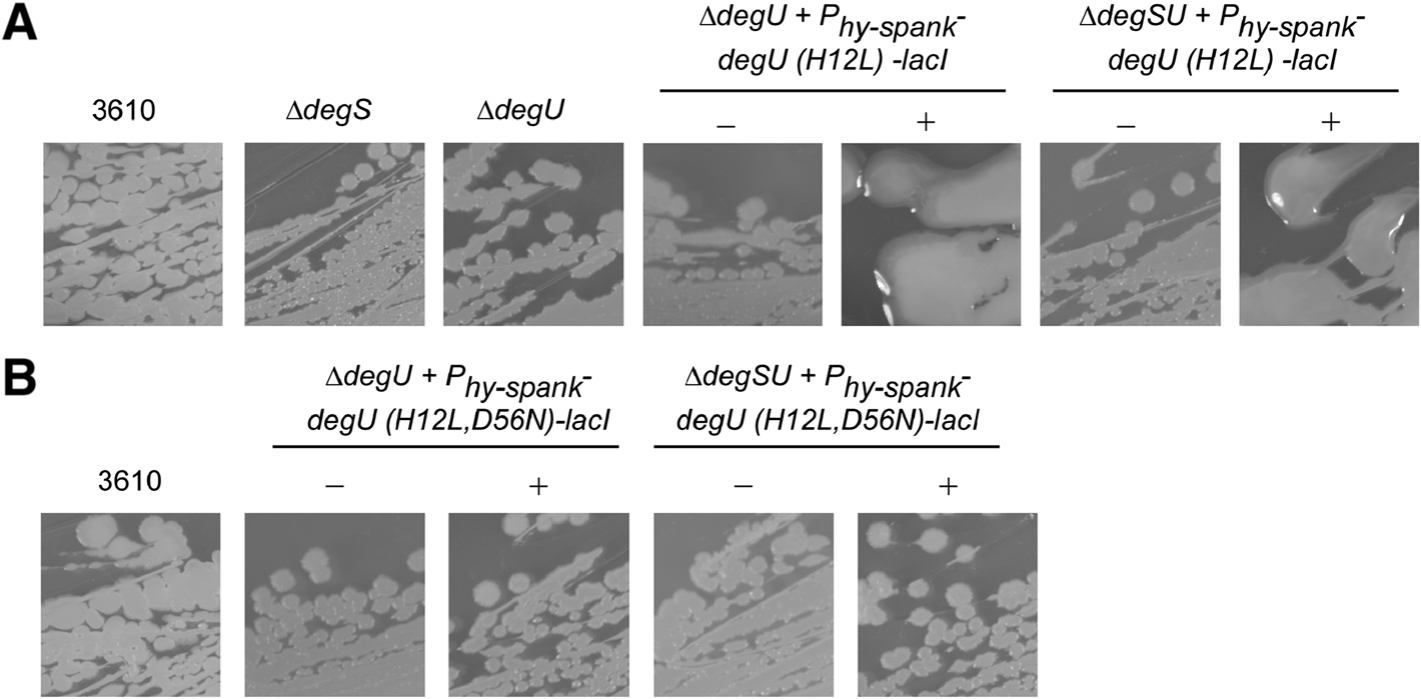
**Production of γ-PGA by strains expressing DegU protein variants. (A)** Colony morphology of 3610 (wild type strain NCIB3610), *degS* (NRS1358), *degU* (NRS1314), *degU* + P_*hyspank*_ *-degU (H12L)-lacI* (NRS1325) and Δ*degSU* + P_*hyspank*_ *-degU (H12L)-lacI* (NRS4419) grown on LB agar plates in the absence (−) or presence (+) of 50 μM IPTG. **(B)** Colony morphology of 3610 (wild type strain), *degU* + P_*hy-spank*_ *-degU (H12L, D56N)-lacI* (NRS4762) and Δ*degSU* + P_*hy-spank*_ *-degU (H12L, D56N)-lacI* (NRS4761) grown on LB agar plates in the absence (−) or presence (+) of 50 μM IPTG. Plates were photographed after 16 h incubation at 37°C.

First, production of the exopolymer γ-PGA was assessed by the presence or absence of a mucoid colony morphology after growth on LB agar [17]. Production of γ-PGA is driven by the protein products of the *pgsB* operon. It is important to keep in mind that while the undomesticated *B. subtilis* NCIB3610 strain has an intact *pgsB* biosynthetic operon on the chromosome, the polymer is not typically synthesised under laboratory conditions [32-34]. However, an increase in the level of DegU ~ P results in enhanced transcription of genes required for the synthesis and export of γ-PGA, culminating in the generation of a mucoid colony morphology [21]. Induction of *degU (H12L)* expression with IPTG in the absence of the native copy of the *degU* gene resulted in a mucoid colony morphology (Figure 1A), as previously reported [17,21]. Induction of *degU (H12L)* in a strain lacking the native copies of both *degS* and *degU* also produced a mucoid colony morphology (Figure 1A). These data indicate that DegU H^12^L is able to stimulate γ-PGA production regardless of whether DegS is present or not.

**Figure 2:**
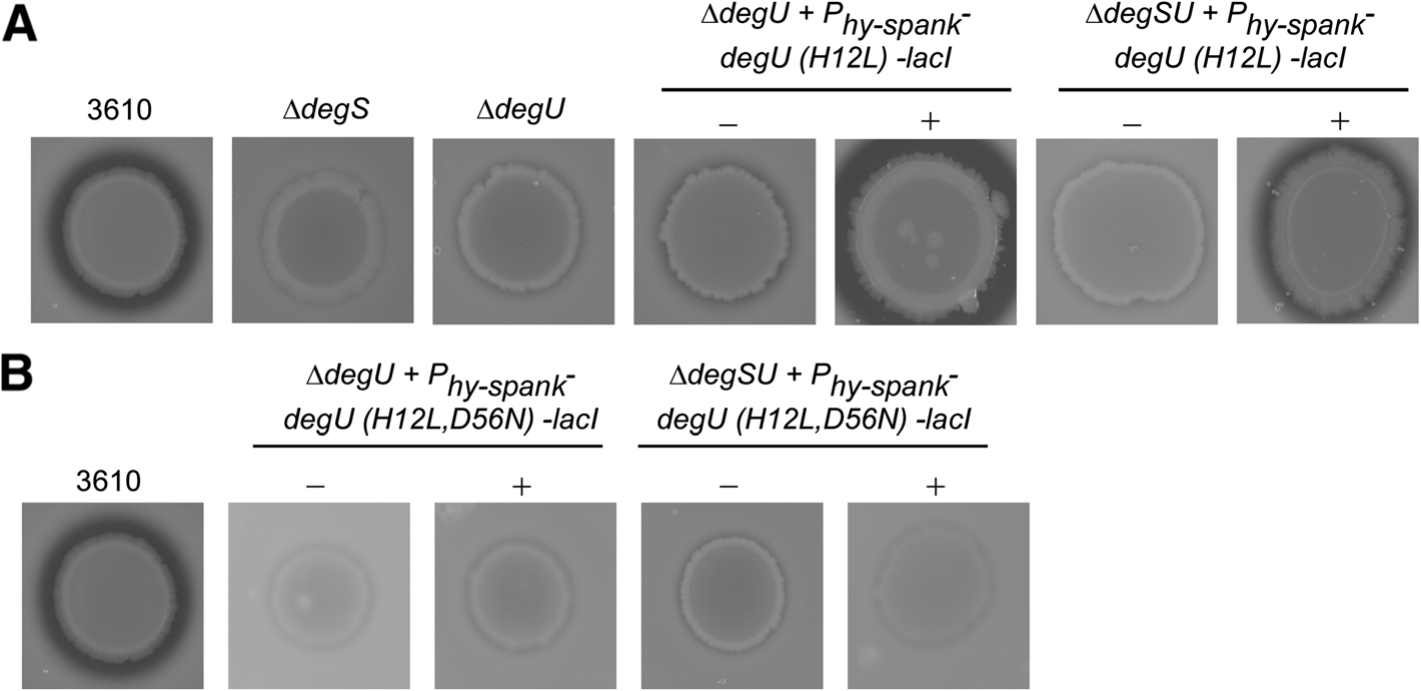
**Analysis of exoprotease production by strains expressing DegU protein variants. (A)** Exoprotease production of 3610 (wild type strain), *degS* (NRS1358), *degU* (NRS1314), *degU* + P_*hyspank*_ *-degU (H12L)-lacI* (NRS1325) and Δ*degSU* + P_*hyspank*_ *-degU (H12L)-lacI* (NRS4419) grown on LB agar plates containing 1.5% milk in the absence (−) or presence (+) of 50 μM IPTG. **(B)** Exoprotease production of 3610 (wild type strain), *degU* + P_*hy-spank*_ *-degU (H12L, D56N)-lacI* (NRS4762) and Δ*degSU* + P_*hy-spank*_ *-(H12L, D56N)-lacI* (NRS4761) grown on LB agar plates containing 1.5% milk in the absence (−) or presence (+) of 50 μM IPTG. Plates were photographed after 16 h incubation at 37°C.

The second assay to measure DegU ~ P activity exploited the fact that high levels of DegU ~ P are required for the production of extracellular proteases (exoproteases) [26,35]. DegU ~ P positively impacts the transcription of several genes involved in protease production, including *aprE*, which encodes the major exoprotease subtilisin [36]. To test if DegU H^12^L could positively control protease production in the absence of *degS*, the *degU (H12L)* strains were grown in liquid culture and spotted on to LB agar plates containing milk as a protease substrate. As shown in Figure 2A, the wild-type strain (3610) shows a zone of clearing around the cells, indicative of exoprotease activity. No such clearing was observed in the absence of either *degS* or *degU* in the otherwise wild-type strain background, consistent with the accepted conclusion that DegU ~ P is essential for exoprotease production [26,35]. Induction of *degU (H12L)* in either the presence or absence of *degS* produced a zone of clearing around the cells, suggesting that DegU H^12^L is active even in a strain that lacks the sensor kinase (Figure 2A). It is noteworthy that the zone of clearing appeared to be reproducibly smaller when *degU (H*^*12*^*L)* was induced in the absence of *degS* when compared to the strain that carried the *degS* coding region. These data are consistent with the conclusion that although DegU H^12^L is active in the absence of DegS, it is unable to reach the same level of activity that is observed in the presence of the kinase.

**Figure 3:**
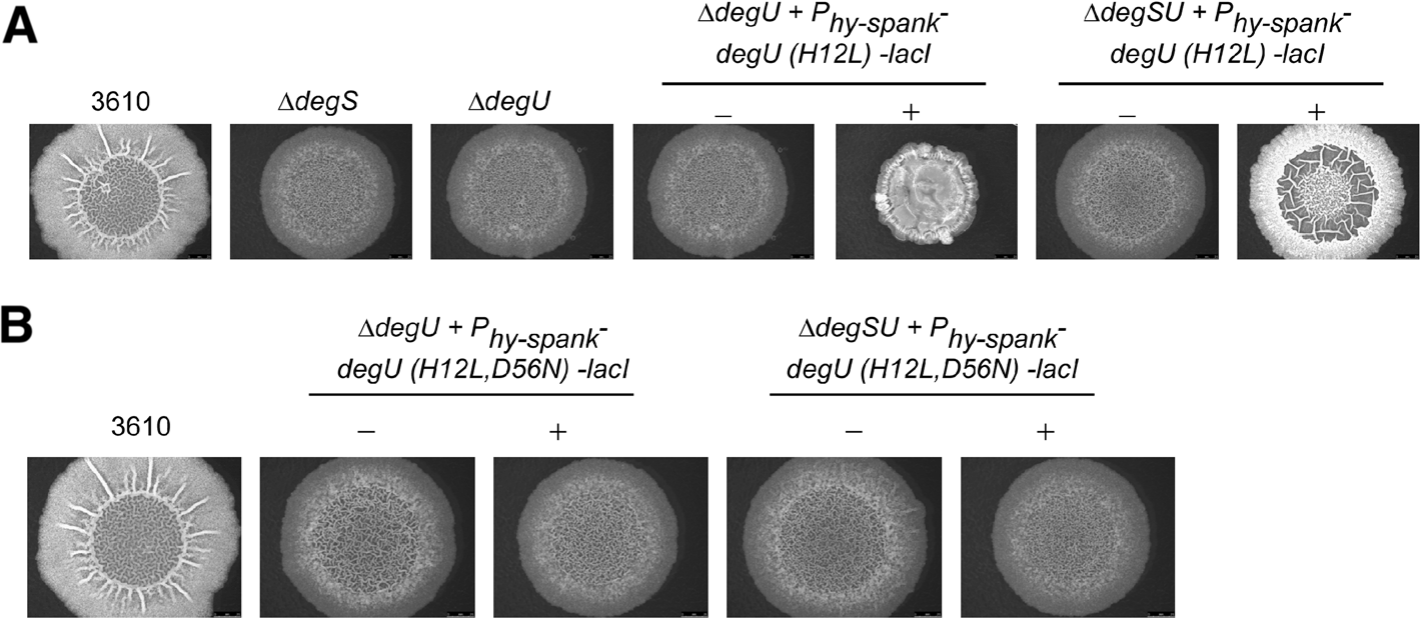
***In vivo*** **macrocolony biofilm development of strains carrying DegU protein variants. (A)** Macrocolony morphology of 3610 (wild-type strain), *degS* (NRS1358), *degU* (NRS1314), *degU* + P_*hyspank*_ *-degU (H12L)-lacI* (NRS1325) and Δ*degSU* + P_*hyspank*_ *-degU (H12L)-lacI* (NRS4419) grown on MSgg agar plates in the absence (−) or presence (+) of 50 μM IPTG. **(B)** Colony morphology of 3610 (wild-type strain), *degU* + P_*hy-spank*_ *-degU (H12L, D56N)-lacI* (NRS4762) and Δ*degSU* + P_*hy-spank*_ *-degU (H12L, D56N)-lacI* (NRS4761) grown on MSgg agar plates in the absence (−) or presence (+) of 50 μM IPTG. All colonies were imaged after 48 h incubation at 30°C.

Third, the ability of DegU H^12^L to influence the morphology of sessile macrocolony biofilm structures was tested (Figure 3A). DegU ~ P is needed for biofilm formation as it indirectly controls transcription of the *bslA* coding region and therefore the formation of a hydrophobic coat surrounding the cells residing within the biofilm [37,38]. However, the level of DegU ~ P needs to be tightly controlled during biofilm formation. At high levels of DegU ~ P, and indeed when DegU H^12^L is used, synthesis of the biofilm matrix is impeded due to indirect repression of the transcription of both the *epsA-O* and *tapA* operons [23] that are required for production of exopolysaccharide and protein components of the matrix, respectively (for a review see [39]). Based on the assumption that DegU H^12^L retained activity in the absence of DegS under biofilm formation conditions two scenarios were postulated: *(i)* if DegU H^12^L reflected wild-type DegU ~ P levels the macrocolony structures would resemble the highly complex and wrinkled three dimensional structures formed by the wild-type or *(ii)* if the level of DegU ~ P was very high, biofilm formation would be repressed and a mucoid morphology would be evident. As expected from previous analyses, the *degS* and *degU* deletion control strains formed colonies lacking structural complexity [18] (Figure 3A). Moreover, expression of *degU (H12L)* in the absence of the native *degU* gene inhibited biofilm formation and produced a mucoid colony [18,23] (Figure 3A). In contrast, expression of *degU (H12L)* in the absence of both *degS* and *degU* resulted in the growth of colonies that showed a wrinkling morphology, albeit distinct from that of the wild-type. These data indicate that in the absence of DegS the activity of DegU H^12^L is sufficient to allow production of the matrix components. In combination these analyses indicate it is reasonable to conclude that DegU H^12^L retains activity in the absence of *degS,* and moreover at the level of induction provided here reaches a level of activity above that seen for the wild-type DegU ~ P in strain NCIB3610 under multiple conditions.

### Phosphorylation of DegU H^12^L is essential for activity

The ability of DegU H^12^L to retain activity in the absence of DegS could be explained by two alternate hypotheses: *(i)* DegU H^12^L is constitutively active or *(ii)* DegU H^12^L can be phosphorylated by an alternative means. While it has been reported that DegU H^12^L is not constitutively active, data to support this statement were not presented and the analysis was performed in a strain background that both lacked SwrA and contained low levels of the DegQ protein that aids phosphotransfer [12]. Thus to distinguish between these conjectures a strain carrying a double mutant *degU (H12L)-degU (D56N)* allele under the control of an IPTG inducible promoter was constructed (Table 1). Expression of this gene produces the DegU H^12^L, D^56^N protein variant, a non-phosphorylatable derivative of DegU H^12^L. If this variant lost activity the data would be consistent with phosphorylation being essential for the activity of the DegU H^12^L protein, i.e. in this scenario the DegU H12L protein would not be constitutively active.

**Figure 4:**
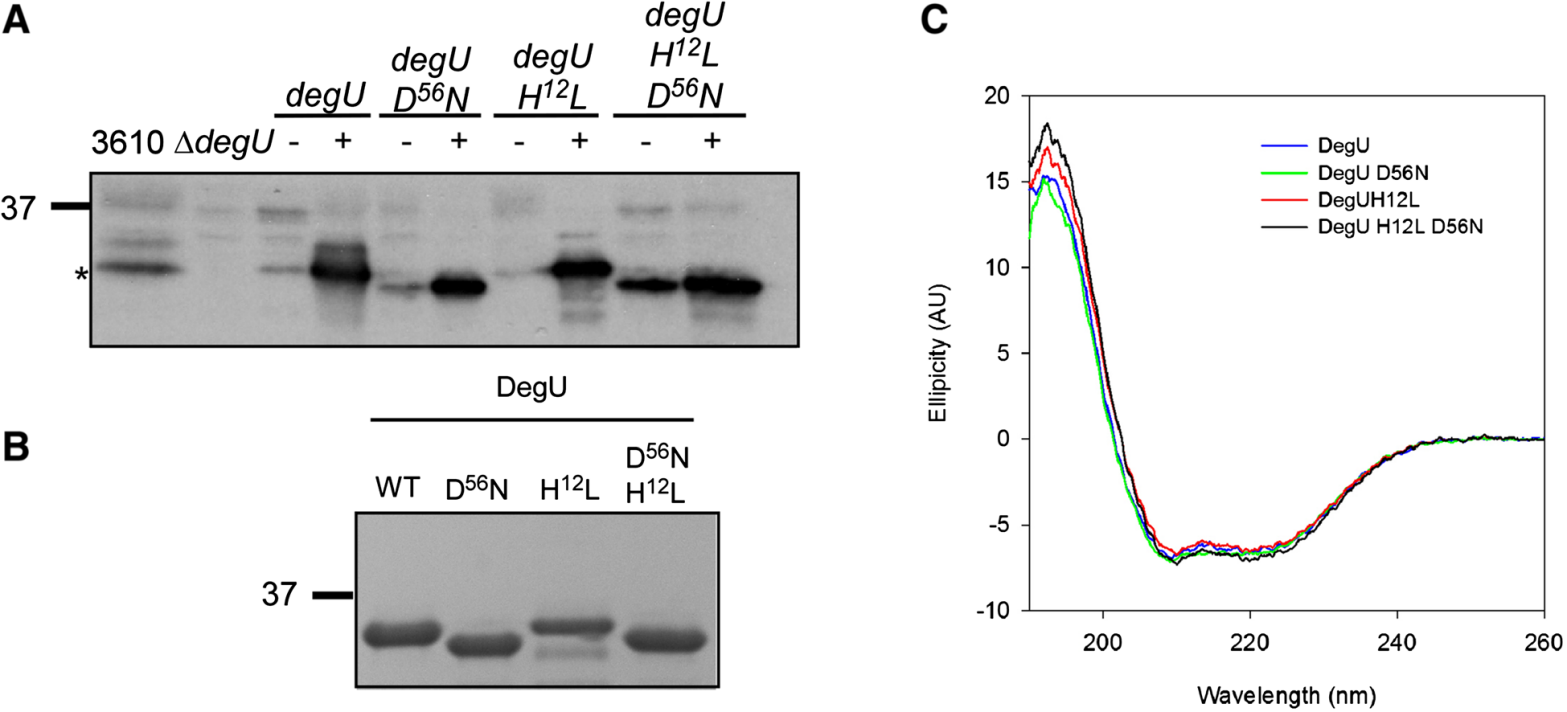
**Analysis of DegU protein variants produced** ***in vivo*** **and in** ***vitro.*** **(A)** Western blot analysis of proteins extracted from 3610 (wild-type strain), *degU* (NRS1314), *degU* + P_*hyspank*_ *-degU (H12L)-lacI* (NRS1326), *degU* + P_*hy-spank*_ *-degU (D56N)-lacI* (NRS1327), *degU* + Phy-spank-*degU* *(H12L)-lacI* (NRS1325) and *degU* + P_*hy-spank*_ *-degU (H12L, D56N)-lacI* (NRS4762), grown in the absence (−) or presence (+) of 50 μM IPTG. The band corresponding to DegU is indicated by an asterisk. The blot was probed with a DegU-specific polyclonal antibody. **(B)** Coomassie stained SDS-PAGE analysis of purified recombinant proteins. Shown are: DegU WT (pNW43), DegU D^56^N (pNW52), DegU H^12^L (pNW54) and DegU H^12^L D^56^N (pNW1072). **(C)** Circular dichroism analysis of the proteins detailed in (B). The plotted spectra are normalised to each other to account for concentration and zeroed at 260 nm after the background spectrum was subtracted.

Initial experiments were undertaken to ensure that introducing two amino acid changes did not impact the production of the DegU protein. To achieve this, proteins were extracted from cell lysates collected from planktonic cultures grown to stationary phase. The samples were separated by SDS-PAGE and probed with a DegU-specific antibody by Western blotting. As shown in Figure 4A a signal could be detected for all DegU protein variants tested, that is wild-type DegU (DegU WT), DegU D^56^N, DegU H^12^L and DegU H^12^L, D^56^N. Therefore mutation of the protein does not impact its production or stability *in vivo*. We noted that both proteins containing the D^56^N mutation migrated at a slightly different apparent molecular mass to that of DegU WT and DegU H^12^L. Given that aspartic acid phosphorylation is an extremely labile modification [25] and unlikely to be visualised on using Western blot analysis, these differences could indicate that mutation of the phosphorylation site alters the conformation of the proteins. Indeed, similar differences in the mobility of DegU and the variant proteins were observed when recombinant protein purified from *E. coli* was analysed by Coomassie staining after SDS-PAGE (Figure 4B). However, circular dichroism experiments revealed that all four protein variants shared similar alpha helix dominated secondary structures with no significant structural differences, at least at the secondary structure level (Figure 4C). In short, introduction of the point mutations did not impact protein production, folding or stability.

Next, assays were undertaken to determine the activity and functionality of the DegU H^12^L, D^56^N protein. As before, DegU ~ P levels were assessed by observation of γ-PGA production (Figure 1B), exoprotease production (Figure 2B) and complex colony formation (Figure 3B) as indicators. In a strain background where the *degU (H12L)-degU (D56N)* coding region was expressed in the absence of native *degU* neither γ-PGA production, protease activity nor complex colony formation was observed. Identical phenotypes were seen when *degU (H12L)-degU (D56N)* was expressed in a strain background lacking both *degS* and *degU*. Therefore both strains produce phenotypes identical to those seen for a *degU* null strain (compare Figures 1A, 2A and 3A with Figures 1B, 2B and 3B). These data demonstrate that the DegU H^12^L, D^56^N protein shows no activity in either the absence or presence of DegS. Thus DegU H^12^L is unlikely to be constitutively active and depends on the phosphorylation of aspartic acid 56 for activity. In combination with our previous findings [17,40], these data demonstrate that DegU can be phosphorylated in the absence of the cognate sensor kinase, DegS.

### DegU can be phosphorylated by acetyl phosphate in the absence of its cognate sensor kinase

**Figure 5:**
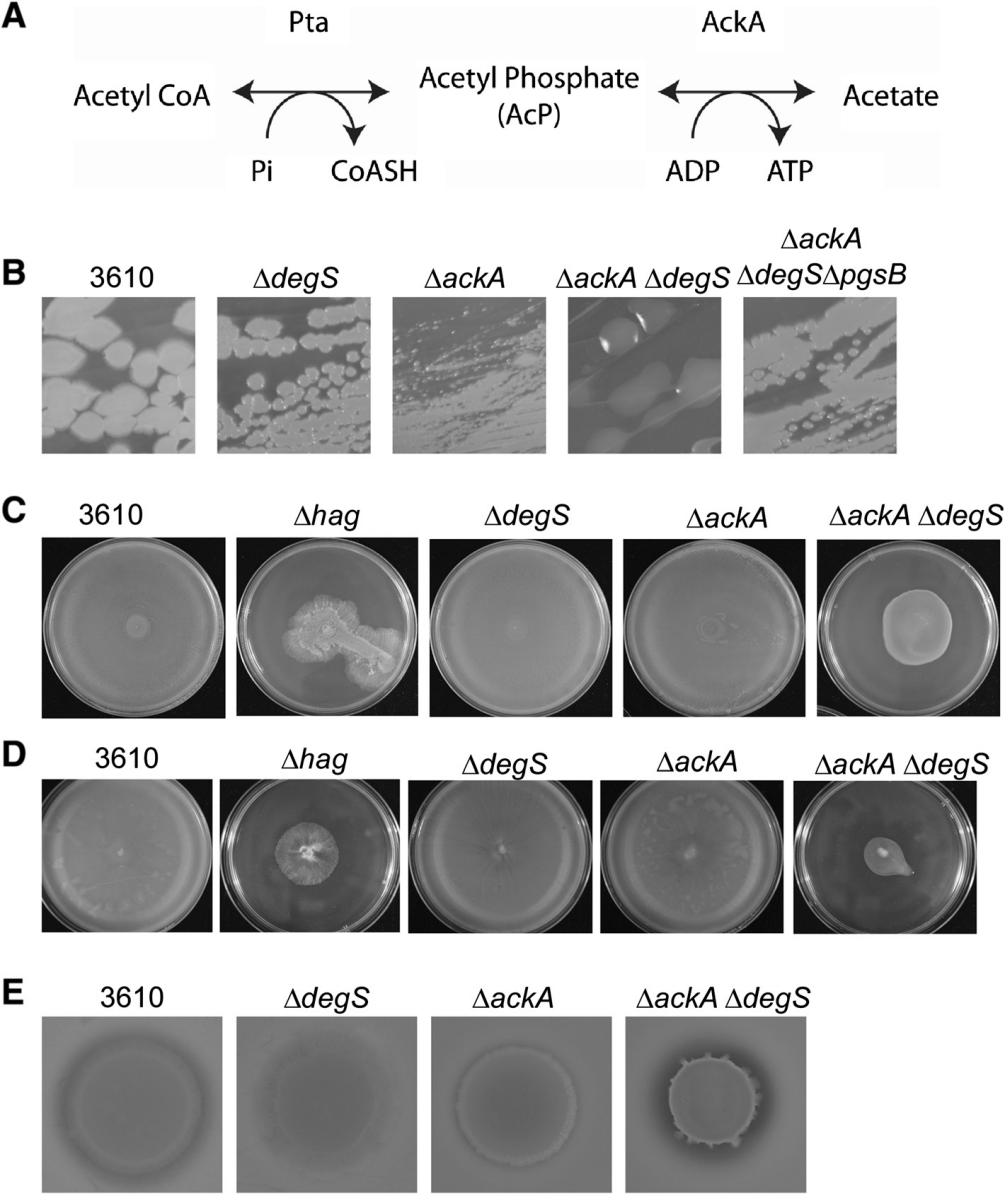
**The impact of elevated AcP levels on DegU ~ P regulated processes. (A)** Schematic diagram of the Pta-AckA pathway **(B)** Colony morphology of 3610 (wild-type), *degU* (NRS1314), *ackA* (NRS4770), *ackA degS* (NRS4771) and *ackA degS pgsB* (NRS4819). Pictures were taken after 16 h incubation at 37°C. **(C)** Photographs of swarm expansion plates taken after 6 h of incubation at 37°C. Shown are 3610 (wild-type), Δ*hag* (DS1677), *degS* (NRS1358), *ackA* (NRS4770), *ackA degS* (NRS4771). **(D)** Photographs of swim expansion plates taken after 16 h incubation at room temperature. Shown are 3610 (wild-type), Δ*hag* (DS1677), *degS* (NRS1358), *ackA* (NRS4770) and *ackA degS* (NRS4771). **(E)** Exoprotease production of 3610 (wild type strain), *degS* (NRS1358), *ackA* (NRS4770) and *ackA degS* (NRS4771) grown on LB agar plates containing 1.5% milk. Plates were imaged after 16 h incubation at 37°C.

Phosphorylation of DegU H^12^L in the absence of *degS* could conceivably be mediated by either a non-cognate sensor kinase, or by a small molecule phosphodonor such as acetyl phosphate (AcP) [41]. As there is precedence for the regulation of response regulators by AcP [42-44], and indeed precedence for the orphan response regulator DegU of *Listeria monocytogenes* to be phosphorylated by AcP [45,46], this mechanism was investigated first. AcP is an intermediate in the acetate dissimilation Pta-AckA (or acetogenesis) pathway (for a review see [47]). Production of AcP is catalysed by phosphotransacetylase (Pta; encoded by the *pta* gene) from Acetyl CoA and inorganic phosphate (Pi) [47]. AcP and ADP are then converted to acetate in a reaction that is catalysed by acetate kinase (AckA, encoded by the *ackA* gene) and also produces ATP (Figure 5A). These reactions are reversible. Acetate is excreted by cells during periods of rapid growth and assimilated when carbon sources become depleted. AcP is used by the cell as a means of storing carbon and phosphate, and has also been reported to act as a global signal by influencing the phosphorylation status of several response regulators [47]. AcP is therefore suggested to act as a link between metabolism and cell signalling [41].

To modulate AcP levels within *B. subtilis* strains the Pta-AckA pathway was manipulated by the genetic disruption of the *ackA* gene [48]. In the absence of *ackA* cells accumulate AcP due to their inability to convert AcP to acetate (Figure 5A). This mutation could therefore be used to test if DegU can be phosphorylated in a strain that lacks *degS* when AcP levels are raised. These experiments utilised native DegU for simplicity and to ensure a physiologically relevant means of assessing the impact of AcP on signal transduction. As shown in Figure 5B the wild-type and Δ*ackA* strains showed a flat colony morphology on LB agar plates (note that deletion of *ackA* affects colony size, likely due to effects on cell growth). However, the double Δ*degS* Δ*ackA* strain exhibited a mucoid colony morphology. This mucoidy was linked to γ-PGA as deletion of the first gene in the γ-PGA biosynthesis operon (namely, *pgsB*) in the Δ*degS* Δ*ackA* background resulted in a non-mucoid colony morphology that resembled that of the wild-type strain. These data indicate that raising the level of AcP in the cell can impact DegU phosphorylation in a Δ*degS* strain. We next tested the physiological response using three other assays; namely swimming and swarming motility and exoprotease production.

In agreement with previous work, motility assays revealed that *degS* was not required for swimming or swarming motility when compared with the wild-type strain (Figure 5C and D) [18]. Similarly deletion of *ackA* failed to impact the motility of *B. subtilis* (Figure 5C and D). In contrast, deletion of both *degS* and *ackA* reduced the ability of *B. subtilis* to both swim and swarm (Figure 5C and D). These data are in line with the hypothesis that in the absence of *degS* the increased concentration of AcP can result in DegU ~ P levels being raised to a point that is sufficient to impede motility.

Finally, the ability of the Δ*degS* Δ*ackA* strain to produce exoproteases was determined using the milk agar plate assay. Figure 5E clearly demonstrates that while deletion of *degS* or *ackA* individually resulted in a lack of exoprotease production, deletion of both *degS* and *ackA* produced a zone of clearing around the cells, suggesting that in this background exoproteases are synthesised, secreted and active. Collectively, these data show that increasing AcP levels by disruption of the *ackA* gene results in an increase in DegU ~ P activity when the sensor kinase *degS* is absent.

### Conclusions

The data presented here show that the response regulator DegU can be modified by AcP in the absence of the cognate sensor kinase, DegS and therefore add DegU to the list of bacterial response regulators that can be modified by AcP [41,49]. These findings elucidate the mechanism underpinning the previous hypothesis that DegU and its variant DegU H^12^L could be phosphorylated by an alternative means [17-19]. In this work a series of phenotypic assays show that the DegU H^12^L protein variant retains its activity in the absence of *degS*. This activity was lost upon mutation of the phosphorylation site at aspartic acid 56, indicating that DegU H^12^L is unlikely to be constitutively active and that the phosphorylation site is essential for its activity (Figures 1B, 2B and 3B). Further experiments were designed to manipulate the level of AcP within the cell by genetic disruption of the *ackA* coding region. Assays confirmed that the native DegU protein could be modified by AcP, as evidenced by *(i)* an increase in γ-PGA production, *(ii)* an increase in protease activity and *(iii)* a reduction in swimming and swarming motility (Figure 5). These findings suggest that an additional layer of regulation exists for DegU, namely the input of metabolic signals that trigger a change in flux through the Pta-AckA pathway. Input of metabolic signals to regulate the DegS-DegU regulatory system might not be surprising given the number of processes that the pathway controls. Given that these effects were observed in the absence of *degS*, this work supports the hypothesis that the phosphatase activity of DegS plays a pivotal role in controlling the phosphorylation status of DegU ~ P [26]. This is perhaps best evidenced by the difference in DegU ~ P regulated phenotypes displayed by the Δ*ackA* and Δ*ackA* Δ*degS* strains (Figure 5). Deletion of *ackA* alone does not impact DegU ~ P phenotypes, suggesting that when DegS is present phosphorylation of DegU by AcP is reversed by the phosphatase activity of DegS. However phosphorylation of DegU via the action of AcP could occur in the wild-type strain if the balance of DegS and DegU within the cell is tipped such that DegU becomes dominant. This could conceivably occur if *degU* is transcribed at a higher rate than *degS*, perhaps when internal promoters within the *degS-degU* operon are activated (for a review see [16]). Finally, it is important to note that a homologue of *degU* gene is encoded within the *L. monocytogenes* genome, but no homologue of *degS* has been identified. In this scenario modification of DegU by AcP is likely to be important for the activity of the response regulator [45].

Previous work suggested that the DegU H^12^L protein variant does not accurately mimic high levels of DegU ~ P due to its inability to interact with SwrA [24]. Indeed, the authors suggested that high levels of native DegU ~ P do not perturb motility, but that these reported effects are due to the use of the *degU (H12L)* allele. It was proposed that the resulting DegU H^12^L protein was unable to interact with SwrA at the *fla/che* promoter, which is needed for the transcription of the flagellar genes, producing a non-motile phenotype [24]. Why DegU H^12^L could not interact with SwrA was not investigated but one possibility could be secondary structure differences. However, circular dichroism experiments presented here (Figure 4C) demonstrate that there are no observable differences between DegU WT and DegU H^12^L at the secondary structural level. It is possible that any structural differences between DegU and DegU H^12^L are only evidenced at the tertiary level, which would not be detected by the circular dichroism experiments presented here. Alternatively, differences may only be seen when the proteins are phosphorylated. The data presented here also suggest that high levels of DegU ~ P (and not just DegU H^12^L) can impede surface motility. This is concluded as raising the level of AcP within the cell to induce phosphorylation of native DegU, impedes both swimming and swarming motility (Figures 5C and D). This effect is only seen in the Δ*ackA* Δ*degS* strain and not in the Δ*ackA* background, suggesting that this phenotype is specific to DegU ~ P and is not due to AcP impacting other aspects of flagellar motility. The differences between our findings and those of Mordini *et al.* could perhaps be explained by the use of different *B. subtilis* strains which, although related, show key differences. For example, the ancestral strain NCIB3610 used in this study carries a large plasmid [34], whereas the *B. subtilis* 168 strain used by Mordini *et al.* lacks this plasmid and, despite carrying an intact *swrA* gene, contains several additional point mutations as a result of genetic manipulations. Further work would be needed to define how mutation of histidine 12 results in an increase in the half-life of DegU ~ P. One possibility is that the H^12^L substitution affects the interaction with DegS and as such decreases its ability to dephosphorylate DegU H^12^L.

Overall, these data suggest that in the absence of the sensor kinase DegS, the response regulator DegU can be modified by AcP. When DegS is present this effect is not seen, indicating that the phosphatase activity of DegS is crucial in regulating the level of DegU ~ P within the cell to prevent unnecessary activation (or inhibition) of downstream target processes. Furthermore, these data show that DegU H^12^L requires phosphorylation for its activity and indicate that DegU H^12^L can mimic the effects of high levels of native DegU ~ P in the ancestral *B. subtilis* NCIB3610 strain.

## Abbreviations

RR: Response regulator
TCS: Two-component signal transduction system
HK: Histidine kinase
AcP: Acetyl phosphate
